# Predicting invasiveness and disease-specific survival in upper tract urothelial carcinoma: identifying relevant clinical tumour characteristics

**DOI:** 10.1007/s00345-019-02760-4

**Published:** 2019-04-23

**Authors:** Camilla Malm, Alexandra Grahn, Georg Jaremko, Bernhard Tribukait, Marianne Brehmer

**Affiliations:** 1 Department of Oncology and Pathology, Karolinska Institutet, Stockholm, Sweden; 2 Department of Urology, Stockholm South General Hospital, Stockholm, Sweden; 3 Division of Urology, Karolinska University Hospital, Stockholm, Sweden; 4 Department of Clinical Pathology and Cytology, Karolinska University Hospital Solna, Stockholm, Sweden; 5 Division of Urology, Department of Clinical Sciences, Danderyd Hospital, Karolinska Institutet, Stockholm, Sweden

**Keywords:** Upper tract urothelial carcinoma, Diagnostics, Staging, Survival, Disease-specific survival, Ureteroscopy, Radical nephroureterectomy

## Abstract

**Purpose:**

The aim of this prospective study was to identify the tumour characteristics that are associated with invasiveness and those that are relevant for disease-specific survival (DSS) in upper tract urothelial carcinoma, UTUC.

**Methods:**

From a prospective consecutive cohort of patients with suspicion of UTUC, those who were diagnosed with UTUC using URS prior to rNU between 2005 and 2012 were included. Tumour characteristics were analysed for prediction of invasiveness and association with DSS. Stages were categorised as superficial (pTa-1 and CIS only) or invasive (≥  pT2). Tumours were graded according to WHO 1999 classification. DSS was analysed regarding possible association with stage, grade, size, multifocality, location, ploidy and rate of proliferation. Associations were tested using Fisher’s exact test, Pearson Chi-square or Cox’s regression. Kaplan–Meier survival curves were constructed.

**Results:**

Forty-five consecutive patients were included, and 43 of them were included in the final analyses because their rNU specimens were available for reassessment. The only tumour characteristics that were significantly associated with stage were tumour grade (*P *< 0.001), DNA ploidy (*P* = 0.045) and rate of proliferation (*P* = 0.004). No association with stage was noted for size, multifocality or location. Grade, stage and rate of proliferation were associated with DSS.

**Conclusions:**

Grade, DNA ploidy and S-phase fraction were the only tumour characteristics associated with stage in our study. However, DNA ploidy was not associated with DSS. The prognostic factors that we identified were tumour grade, stage, and S-phase fraction.

## Introduction

Balancing preservation of renal function against optimisation of oncological treatment in patients with upper tract urothelial carcinoma (UTUC) is challenging, and the guidelines developed by the European Association of Urology (EAU) have addressed this issue by dividing UTUC into high- and low-risk disease [1]. Evidence of renal insufficiency as an independent risk factor for mortality and cardiovascular disease [2] strengthens the incentive to reserve radical nephroureterectomy (rNU) for high-risk patients. Performing rNU on superficial low-grade tumours is actually overtreatment in many cases [3]. Considering disease-specific survival (DSS), organ-sparing treatment may represent a good option in patients with low-risk UTUC [1, 4–7]. Being able to differentiate between low- and high-risk UTUC is of the utmost importance to aid treatment decisions, and accurate risk stratification is crucial in this context. A reliable model for preoperative identification of stage and organ confinement is essential for choice of treatment modality. Preoperative prognostic models based on retrospective data have been proposed for identification of non-organ-confined UTUC [8, 9], and these approaches suggest that tumour grade and hydronephrosis on imaging are key factors predicting invasiveness. However, imaging, even computed tomography urography (CTU), offers insufficient accuracy for staging of UTUC [10, 11]. For optimal diagnostics, radiological investigations should be combined with ureterorenoscopy (URS) and analysis of cytopathological samples [10, 12].

Stage and grade seem to be the most important prognostic factors in UTUC [1, 3, 5, 7, 13, 14]. Direct staging using biopsies is not possible [15], because biopsies must be small and superficial to avoid ureteral perforation and risk of tumour seeding [16, 17]. Tumour heterogeneity may be an important factor complicating correct grading from small biopsies. This problem persists despite promising novel diagnostic methods such as confocal laser endomicroscopy or optical coherence tomography [18]. Good correlation between grade and stage has been observed in some studies [17, 19].

Regarding grading, voided urine cytology and focal cytology (taken as drip cytology) have been reported to have sensitivity as low as 20% [20], whereas barbotage cytology has proven to be highly sensitive [21]. Furthermore, several studies have noted that it is difficult to achieve correct grading of endoscopic samples [22, 23], but that diagnostic accuracy can be improved by examining both barbotage cytology and biopsy specimens [12, 21]. Nevertheless, it is clear that direct staging of UTUC has limitations.

The aim of this prospective study was to investigate UTUC to identify the tumour characteristics that are associated with invasiveness and those that are relevant for DSS.

### Patients and methods

The study was approved by the Regional Ethical Review Board and was performed in accordance with the Declaration of Helsinki. Informed consent was obtained from all patients.

From a prospective consecutive cohort of patients with suspicion of UTUC, those who were diagnosed with UTUC using URS prior to rNU between 2005 and 2012 were included in the present study. Tumour characteristics were analysed for prediction of invasiveness and association with DSS. All rNUs were performed within 1 month after URS. rNU was offered to all patients with UTUC lacking absolute contraindications, as recommended in the EAU guidelines at that time. All patients had M0 disease. The protocol for diagnostic URS and collection of samples has previously been described [21]. In short, at URS, focal barbotage specimens were obtained for cytology and DNA ploidy. Flow cytometry DNA ploidy in endoscopic barbotage samples have previously been proven comparable to ploidy in rNU specimens [21]. Biopsies were taken from suspicious lesions, using Piranha™ 3 Ch ureteroscopic biopsy forceps (Boston Scientific Nordic AB, Helsingborg, Sweden).

Tumour size (surface diameter) was visually assessed at URS and also measured in rNU specimens. Tumour stage, grade, ploidy, and rate of proliferation [24] were evaluated in subsequent rNU specimens (paraffin-embedded tissue blocks).We also analysed association with stage for the following parameters: grade, size, multifocality, location, flow cytometry of DNA ploidy, and rate of proliferation (proportion of cells in S-phase of cell cycle). Rate of proliferation was also tested for possible association with tumour grade. Ploidy was categorised as diploid (diploid and tetraploid) or aneuploid (non-tetraploid aneuploid). Surgical technique of rNU varied, one-third of the operations were performed laparoscopically, whereas the others were performed with open surgery. In the majority of the cases the bladder cuff was removed by extravesical open approach.

Stage was categorised as superficial (pTa-1 [± CIS] or CIS only), or invasive (≥  pT2, ± CIS). Tumours were graded according to the WHO 1999 classification [25], which is used as the standard in our region, because it is considered to be more useful clinically and also to offer higher resolution and more accurately predict invasiveness compared with the WHO 2004 classification [5, 13]. The TNM/UICC system 2002 [26] was used for tumour staging. Ploidy and rate of proliferation in rNU specimens were determined by flow cytometry. Tumour size of exophytic tumours was categorised by a surface diameter smaller or larger than 20 mm. rNU specimens and focal samples were reassessed by a single specialised pathologist.

DSS in April 2018 was analysed regarding possible association with stage, grade, size, multifocality, location, ploidy and rate of proliferation.

### Statistical analysis

Associations and hazard ratios were tested using Fisher’s exact test, Pearson Chi-square or Cox’s regression. Kaplan–Meier survival curves were constructed. Ninety-five percent confidence intervals (CIs) were calculated, and a level of 0.05 was considered significant. The statistical analyses were performed using SPSS 23.0 and Microsoft Excel for Mac 2011 (14.3.9). A professional statistician was consulted.

## Results

45 consecutive patients were prospectively included, and 43 of them were included in the final analyses because their rNU specimens were available for reassessment. 26 of these 43 patients had exophytic superficial tumours, 12 had invasive tumours, and 5 had CIS only. Patient and tumour characteristics are listed in Table 1.

**Table 1:** Patient and tumour characteristics of 43 patients who underwent nephroureterectomy after ureterorenoscopy

Age at diagnosis (years)	
Mean	68.8
Median (range)	68 (34–89)
Sex	
Female, number (%)	11 (26)
Male, number (%)	32 (74)

	Number (%)
Reason for investigation	
Malignant cells in bladder/ureter cytology	5 (11.6)
Macroscopic finding at cystoscopy (ostium)	4 (9.3)
Pain	2 (4.7)
Macroscopic haematuria	23 (53.5)
Referred with highly suspected UTUC	1 (2.3)
X-ray finding	7 (16.3)
Other	1 (2.3)
History of bladder cancer	
Number of patients (%)	15 (35)
Secondary bladder cancer	
Number of patients (%)	6 (14)
Cause of death	
Urothelial carcinoma	10
Other	6
Tumour location, *n* (%)	
Renal pelvis	23 (53)
Ureter	13 (31)
Pelvis and ureter	4 (9)
No visible pathology at URS	3 (7)^a^
Tumour focality	
Unifocal	24 (55.8)
Multifocal	16 (37.2)
No visible tumour at URS	3 (7)^a^

		Number (%)		Number (%)
Tumour stage	Superficial	26 (60.5)	Ta	16 (37.2)
	pTa-pT1		Ta + CIS	2 (4.7)
			T1	7 (16.3)
			T1 + CIS	1 (2.3)
	CIS only	5 (11.6)	CIS only	5 (11.6)
	Invasive > PT1	12 (27.9)	T2	3 (7.0)
			T2 + CIS	2 (4.7)
			T3	5 (11.6)
			T3 + CIS	1 (2.3)
			T4	1 (2.3)

	Number (%)
Tumour grade WHO classification 1999	
G1	10 (23.3)
G2	13 (30.2)
G3	20 (46.5)
Tumour surface diameter^b^	
≤ 20 mm	19 (44.2)
> 20 mm	16 (37.2)
CIS only	5 (11.6)
Tumour ploidy	
Diploidy (diploid and tetraploid)	22 (51.2)
Aneuploidy (non-tetraploid aneuploid)	21 (48.8)
Combined stages and grades **number**	
Superficial (pTa, pT1 ± CIS) **26**	G1 **10**
	G2 **12**
	G3 **4**
CIS only	G3 **5**
Invasive (pT2-4 ± CIS) **12**	G1 **0**
	G2 **1**
	G3 **11**

	Mean, % (95% CI)	Median, % (range)
Rate of proliferation (proportion of cells in S-phase of the cell cycle), overall mean 7.156		
Superficial (pTa, pT1 ± CIS) *N *= 26	6.0 (4.0–8.1)	3.8 (0.4–16.6)
CIS only *N *= 5	3.3 (0.3–6.3)	2.4 (1.2–6.7)
Invasive (pT2-4 ± CIS) *N *= 12	11.2 (8–14.3)	11.7 (2.6–21.8)
Grade 1	2.2 (1.1–3.3)	2 (0.4–5.7)
Grade 2	6.5 (3.8–9.3)	5.4 (2.1–16.6)
Grade 3	10.0 (7.6–12-5)	11 (1.2–21.8)

^a^All were CIS only

^b^Unknown size in three patients

S-phase was statistically significantly different between exophytic superficial and invasive UTUC (*P* = 0.011), as well as between CIS only and invasive tumours (*P* = 0.011). However, no difference was found between exophytic Ta-T1 tumours and CIS, (*P* = 0.482) (Table 1).

A statistically significant association was also found between ploidy and grade (*P* < 0.001) and rate of proliferation (S-phase) and grade (G1: 2.2% [95% CI 1.1–3.3], G2: 6.5% [95% CI 3.8–9.3], G3: 10.0 [95% CI 7.6–12.5]). The only tumour characteristics that were significantly associated with stage, superficial (pTa-1, CIS) or invasive (> T1) were tumour grade (*P *< 0.001), DNA ploidy (*P* = 0.045) and rate of proliferation (*P *= 0.002); that is, no association with stage was noted for tumour size, multifocality or location (Table 2).

**Table 2:** Tumour characteristics and their association with stage (pTa-1 and CIS or > T1)

	Statistical significance
Grade	*P* < 0.001, significant
DNA ploidy	*P* = 0.045, significant
Proportion of cells in S-phase of the cell cycle	*P* = 0.004, significant
Size, surface diameter	*P* = 0.78, not significant
Multifocality	*P* = 0.18, not significant
Location of tumour (renal pelvis or ureter)	*P* = 0.31, not significant

**Fig. 1:**
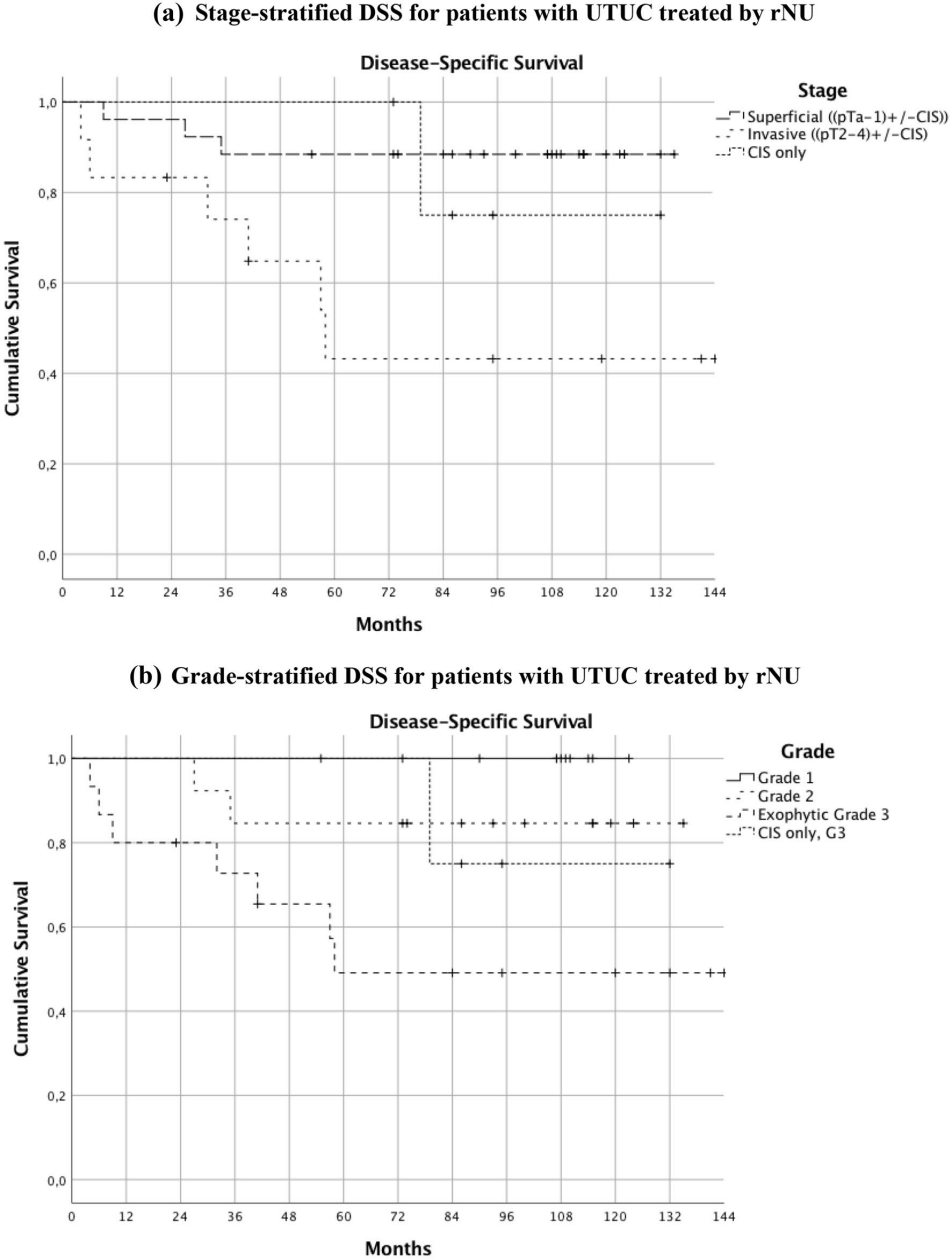
Kaplan–Meier curves of disease-specific survival stratified by tumour stage and grade. **a** Stage-stratified DSS for patients with UTUC treated by rNU. Kaplan–Meier curves of disease-specific survival stratified by tumour stage. 5-year disease-specific survival (60 months) was 88% for superficial tumours, 50% for invasive tumours, 100% for CIS only, and 79% in total. Hazard ratio for DSS in invasive compared with exophytic superficial UTUC was 6.12, *P *= 0.011. Hazard ratio for DSS in exophytic superficial compared to CIS was 1.606, *P* = 0.682 (not significant). **b** Grade-stratified DSS for patients with UTUC treated by rNU. Kaplan–Meier curves of disease-specific survival stratified by tumour grade. 5-year disease-specific survival (60 months) was 100% for G1, 85% for G2, 53% for exophytic G3, 100% for CIS only and 79% in total. Overall log rank was 9.618, *P *= 0.022

Grade, stage and rate of proliferation were the only tumour characteristics that were associated with DSS (*P* = 0.044, 0.023 and 0.006, respectively). Associations between DSS and location (*P* = 0.716), multifocality (*P* = 0.191), tumour size (*P* = 0.719), ploidy (*P* = 0.126), and history of bladder cancer (*P* = 0.866) were not statistically significant. At a median follow-up time of 95 months (range 4–144 months), 16/43 patients had died: 10 from urothelial carcinoma and six from other causes. At that time, DSS for all patients was 77%. Stratified by stage, DSS was 88% for superficial tumours (pTa-1 ± CIS), 80% for CIS only, and 50% for invasive tumours. Stratified by grade, DSS was 100, 85, and 53% for G1, G2, and exophytic G3, respectively. There was a 6.12 times higher risk of dying in invasive UTUC compared to superficial exophytic UTUC, *P* = 0.011. Kaplan–Meier survival curves for stages and grades are shown in Fig. 1. For superficial UTUC (exophytic or CIS) DSS decreased by 25% for every percent increase in rate of proliferation (S-phase) (*P* = 0.027). The proportion of cells in S-phase was significantly higher in invasive tumours than in superficial (*P* = 0.002) but there was no statistically significant decrease in DSS for increasing rate of proliferation in invasive UTUC (*P* = 0.969).

## Discussion

Grade, DNA ploidy and rate of proliferation (S-phase) were the only parameters that predicted invasiveness, of which grade and rate of proliferation were the strongest. Extra caution should be observed when considering G2 tumours, because it is extremely difficult to predict the stage of such lesions based on endoscopic samples, as noted in our analysis as well as in other studies [8, 17, 19, 22, 27–29]. The proportion of invasive G2 tumours in the specimens we investigated was 15% (2/13), which is clearly smaller than the proportions of 28–45% reported by other authors [6, 17, 22, 27]. This suggests that G2 tumours represent a heterogeneous group, and hence a better method is needed to predict invasiveness in this subset. Factors that plausibly affect the mentioned difference are interobserver variability in UTUC pathology evaluation and tumour heterogeneity. Superficial and low-grade tumours are overrepresented in our investigation compared with other reports, which might be explained by the main inclusion criterion we used: patients were to have had URS prior to rNU due to suspicion of UTUC. Some patients with “obvious” findings on imaging were sent straight to rNU during the years covered by our study. Also, our investigation was initiated before organ-sparing treatment was introduced for all patients with what is now defined as low-risk UTUC. Consequently, the present cohort consisted of nearly equal numbers of G1–G3 tumours.

Overall in our study, it was more likely for superficial tumours to be diploid and invasive tumours to be aneuploid. Although there was a strong association between stage and ploidy in general, ploidy could not be used to distinguish between superficial and invasive G2 tumours. The clinical difficulty in this context is illustrated by the following: the two G2 tumours in our material, which would not have been suitable for organ-sparing treatment because they were stage pT2 and pTa + CIS, respectively, were actually diploid. Rate of proliferation was useful in assessing invasiveness, nevertheless, considering the small number of patients with invasive G2 tumours in our investigation, it is impossible to draw any far-reaching conclusions about G2 disease and/or rate of proliferation. CIS had a low rate of proliferation, which may be consistent with slow progression of the disease, as also indicated by the only death in a patient with CIS occurring at 79 months after diagnosis. The survival rates in our study are comparable to those reported by Holmäng and Johansson [13].

Rate of proliferation was strongly associated with DSS. Since rate of proliferation can be analysed from URS barbotage samples [21] it may be a valuable tool to predict high-risk UTUC. However, the number of patients in this study are too few to calculate a cut-off value of rate of proliferation for superficial and invasive UTUC for clinical use. Risk of dying in UTUC with increasing rate of proliferation seems to reach a plateau at a level below the range of invasive disease, but this needs further exploration.

Despite a high proportion of both multifocal tumours and tumours > 20 mm in our material, we found no statistically significant association between stage and multifocality, location, or surface diameter. The lack of correlation between stage and tumour size might be explained by an inappropriate categorisation of size resulting in loss of resolution. In short, size was categorised as CIS or exophytic tumours smaller than or larger than 20 mm, based on risk stratification in the EAU guidelines [1], and 14/26 (54%) of the superficial tumours in our cohort were larger than 20 mm. Evidence regarding the role of tumour size is conflicting, and most studies report size in categories and with different cut-off limits, which makes it difficult to compare results. Our findings agree with those obtained by Villa et al. [7], who questioned the usefulness of tumour size but underlined the importance of grade. On the other hand, Milenkovic-Petronic et al. [30] used a cut-off of 30 mm and found a statistically significant association between stage and size. Some studies have also investigated the impact of size on survival. Notably, both Holmäng and Johansson [13] and Simone et al. [14] observed that larger size was associated with worse outcome, whereas we found no such correlation. Correct grading is more important than a strict size cut-off when selecting treatment modality, because grade is a better predictor of invasion and survival.

## Conclusion

Grade, DNA ploidy and S-phase fraction were the only tumour characteristics associated with stage in our study. However, DNA ploidy was not associated with DSS. The prognostic factors that we identified were tumour grade, stage, and S-phase fraction. Our findings suggest that correct tumour grading plays a crucial role in the diagnostics of UTUC. To further improve preoperative risk stratification, future research should focus on improved methods for correct grading and identification of other reliable markers of high-risk disease, cell proliferation and others, especially with regard to G2 tumours.
